# Baseline Oral Microbiota Richness Is Associated with Training-Induced Improvements in Relative Handgrip Strength in Older Adults

**DOI:** 10.3390/nu18142386

**Published:** 2026-07-22

**Authors:** Javier Conde-Pipó, Maria Leyre Lavilla-Lerma, Alexander Achalandabaso-Ochoa, Tomás Conde-Rienda, Miguel Mariscal-Arcas, Antonio Martínez-Amat

**Affiliations:** 1 Department of Health Sciences, Faculty of Health Sciences, University of Jaén, 23071 Jaen, Spain; jconde@ujaen.es (J.C.-P.); llavilla@ujaen.es (M.L.L.-L.); aaochoa@ujaen.es (A.A.-O.); amamat@ujaen.es (A.M.-A.); 2 Health Science and Nutrition Research (HSNR, CTS-1118), Department of Nutrition and Food Science, University of Granada, Campus of Cartuja s/n, 18071 Granada, Spain; tomasconde@correo.ugr.es; 3 Study Group on Physical Activity (CTS-026), Department of Health Sciences, Faculty of Heath Sciences, University of Jaén, 23071 Jaen, Spain; 4 Instituto de Investigación Biosanitaria de Granada (ibs.GRANADA), 18012 Granada, Spain

**Keywords:** oral microbiota, aging, exercise responsiveness, handgrip strength, microbial richness

## Abstract

**Background:** Considerable inter-individual variability exists in exercise-induced adaptations among older adults. Although microbial ecosystems have been linked to muscle function and physical performance, the role of the oral microbiota in exercise responsiveness remains unclear. **Objective:** To explore whether baseline oral microbiota characteristics are associated with training-induced changes in relative handgrip strength (rHGS) in older adults. **Methods:** This preliminary exploratory longitudinal study included 18 community-dwelling older adults who completed a 16-week supervised exercise intervention. Oral microbiota composition was assessed at baseline using 16S rRNA gene sequencing. Participants were classified as responders when ΔrHGS was >0 and as non-responders when ΔrHGS was ≤0; this operational threshold did not account for measurement error or clinically meaningful change. Associations between baseline microbiota variables and ΔrHGS were examined using group comparisons, Spearman correlations, FDR correction, and exploratory linear regression models. **Results:** Responders showed higher baseline bacterial genus richness than non-responders at the nominal level (86.15 ± 8.90 vs. 71.60 ± 13.32; *p* = 0.023), although this difference did not remain significant after FDR correction (*p*FDR = 0.069). Baseline richness was positively associated with ΔrHGS (ρ = 0.578, *p* = 0.012, *p*FDR = 0.036) and remained associated with ΔrHGS in exploratory sensitivity models. Genus-level findings did not remain significant after FDR correction and were interpreted as exploratory candidate signals. **Conclusions:** In this preliminary cohort, greater baseline oral microbiota richness was associated with larger improvements in rHGS after exercise training. These hypothesis-generating findings require confirmation in larger studies with functional microbiome assessment before causal or predictive interpretations can be made.

## 1. Introduction

Population ageing has become one of the major demographic challenges worldwide [1]. Increased life expectancy has resulted in a growing proportion of older adults, making the preservation of functional independence and quality of life a public health priority [2,3,4].

Regular physical activity and adequate nutrition are widely recognized as key determinants of healthy ageing [5]. Among the various components of physical fitness, muscle strength has received particular attention due to its close relationship with mobility, disability risk, and mortality in older adults [6]. Handgrip strength (HGS) is considered a practical and reliable marker of overall muscle strength and general health status [7]. More recently, relative handgrip strength (rHGS), expressed in relation to body size parameters such as body mass index, has been proposed as a potentially more informative indicator of functional status than absolute strength alone, particularly in ageing populations [8,9,10].

Although exercise training is one of the most effective interventions for improving muscle function in older adults [11], considerable inter-individual variability has been reported in the adaptations achieved following similar exercise programs, with some individuals experiencing substantial improvements, whereas others show only modest or limited changes despite comparable training exposure [11,12]. This variability suggests that biological factors beyond the exercise stimulus itself may influence the adaptive response [11,13]. However, the mechanisms underlying this variability remain incompletely understood, highlighting the need to identify factors associated with greater responsiveness to exercise interventions [14].

Among the biological factors that may contribute to this variability, the gut microbiota has been proposed as a potential modulator of muscle health and physical function [15]. This concept is supported by the microbiota–gut–muscle axis, through which microbial composition may influence skeletal muscle via systemic inflammation, immune regulation, energy metabolism, insulin sensitivity, and the production of microbial metabolites such as short-chain fatty acids [16,17]. In older adults, recent evidence has linked gut microbial diversity and taxonomic composition with muscle strength, physical performance, frailty, and sarcopenia [18]. Although the mechanisms involved have not been fully established, these findings suggest that the microbiota may be one of the biological factors contributing to variability in exercise-induced adaptations.

Although most microbiota–exercise research has focused on the gut, the oral microbiota may also be relevant to exercise adaptation. The oral cavity harbors more than 700 microbial species and constitutes one of the most diverse microbial ecosystems in the human body [19,20]. Beyond its traditional study in relation to oral diseases, the oral microbiota is increasingly recognized as a distinct microbial ecosystem with systemic immune, inflammatory, metabolic, and vascular interactions with the host [20,21,22,23,24,25]. In older adults, recent reviews indicate that ageing is associated with changes in oral microbial diversity and composition, and that oral microbial dysbiosis has been linked to frailty, sarcopenia, and systemic health [20,21,24,25]. Studies in physically active individuals and athletes have reported oral microbial profiles that differ from those of sedentary controls, including differences in genera such as *Rothia*, *Veillonella*, *Gemella*, and *Streptococcus* [19,26,27]. Furthermore, exercise interventions have been associated with changes in oral microbial richness and taxonomic composition, including in older adults [27,28,29,30]. Nevertheless, current evidence remains limited and heterogeneous, and it is still unclear whether baseline oral microbiota characteristics are associated with variability in training-induced adaptations [28].

One potential mechanism linking the oral microbiota with exercise physiology is the enterosalivary nitrate–nitrite–nitric oxide pathway, in which nitrate-reducing oral bacteria may contribute to nitric oxide bioavailability, a molecule involved in vascular function, oxygen delivery, and exercise performance [22,31]. Oral nitrate-reducing capacity has also been positively associated with peak oxygen uptake and peak power output in healthy adults, suggesting that oral microbial activity may be related to physiological processes relevant to exercise performance [32]. Thus, this pathway provides biological plausibility for a relationship between oral microbial communities and exercise-related physiology, but should be interpreted as one possible mechanism within a broader framework that also includes immune, inflammatory, and metabolic host-microbe interactions [31,33].

Therefore, this exploratory study aimed to investigate whether baseline oral microbiota characteristics were associated with changes in relative handgrip strength following a supervised exercise intervention in older adults. Specifically, we distinguished bacterial genus richness, as an overall ecological feature of the oral microbiota, from genus-level relative abundances, as taxonomic features, and examined their associations with the magnitude of change in rHGS. We hypothesized that greater baseline bacterial genus richness, together with selected genus-level abundance patterns, would be associated with larger changes in rHGS. Accordingly, the study was conceived as hypothesis-generating, aimed at identifying candidate associations to inform future research rather than validating oral microbiota-based biomarkers or predictive models.

## 2. Materials and Methods

### 2.1. Study Design

A preliminary longitudinal study was conducted in community-dwelling older adults undergoing a supervised exercise training program. The study was designed to investigate the relationship between baseline oral microbiota characteristics and individual changes in rHGS following the intervention. The study was approved by the Human Research Ethics Committee of the University of Jaén (OCT.18/4.PRY) and was conducted in accordance with the Declaration of Helsinki. All participants provided written informed consent before enrolment. The study was registered at ClinicalTrials.gov (NCT05220670).

### 2.2. Participants

Participants were recruited from community-based physical activity programs in southern Spain. Eligibility criteria included being 65 years of age or older and maintaining independence in activities of daily living. Individuals were excluded if they presented contraindications to exercise participation, medical conditions affecting salivary production, antibiotic treatment during the month prior to saliva collection, unstable cardiovascular disease, severe neurological disorders, or any other condition that could interfere with the intervention or assessments. Participants already engaged in structured exercise programs were also excluded. These criteria were established to minimize potential confounding factors affecting both physical performance and oral microbiota composition.

Of the 28 participants initially enrolled, 10 were excluded from the final analysis, all of whom were men: nine did not complete the exercise training program, and one could not be profiled for baseline oral microbiota. Consequently, the final analytical sample included 18 participants with both a valid baseline oral microbiota profile and complete pre- and post-intervention rHGS data.

### 2.3. Anthropometric Assessment

Baseline demographic and anthropometric data were collected before the intervention by trained personnel following standardized procedures. Age and sex were recorded for all participants. Height was measured to the nearest 0.1 cm using a stadiometer (T201-T4 ASIMED, Barcelona, Spain), and body mass was assessed to the nearest 0.1 kg using a calibrated digital scale (Tefal, Rumilly, France). Body mass index (BMI) was calculated as body mass divided by height squared (kg/m^2^). Participants were measured barefoot and wearing light clothing [5].

### 2.4. Exercise Intervention

Participants completed a supervised exercise program lasting 16 weeks, consisting of two 60 min sessions per week (32 sessions in total). Training was performed on cycle ergometers and supervised by qualified exercise professionals. Each session included a 10 min warm-up, a 40 min aerobic exercise phase, and a 10 min cool-down period. Exercise intensity was prescribed relative to each participant’s maximal heart rate and maintained within a moderate-to-vigorous range (approximately 70–80% of maximal heart rate). Heart rate was continuously monitored using a Polar Team Pro system (Polar Electro Oy, Kempele, Finland) to ensure compliance with the prescribed intensity. Only participants attending at least 80% of the scheduled sessions were considered eligible for the present analysis. The standardized intervention enabled the evaluation of inter-individual variability in rHGS responses according to baseline oral microbiota characteristics.

### 2.5. Handgrip Strength

Muscle strength was assessed using a digital handgrip dynamometer (TKK 5101 Grip-D; Takei Scientific Instruments Co., Ltd., Tokyo, Japan). Participants performed the test in a standardized standing position with the arm fully extended alongside the body. Three maximal attempts were performed using the dominant hand, with a 30 s rest period between trials. The highest value obtained was used for analysis. This standardized protocol was used to reduce within-session measurement variability. Handgrip dynamometry is considered a valid and reliable method for assessing muscle strength in older adults [34,35].

Relative handgrip strength (rHGS) was calculated as handgrip strength divided by body mass index (BMI) [8,10,36]. The primary outcome was the change in relative handgrip strength following the intervention (ΔrHGS), calculated as the difference between post-intervention and baseline values. Participants were subsequently classified as responders or non-responders according to the direction of change in rHGS. Individuals showing a positive change in rHGS (ΔrHGS > 0) were classified as responders, whereas those showing no improvement or a reduction in rHGS (ΔrHGS ≤ 0) were classified as non-responders. This operational classification was used to explore whether baseline oral microbiota characteristics differed according to subsequent training response. However, it was not intended to represent a clinically validated responder definition, as no study-specific standard error of measurement, minimal detectable change, or clinically meaningful threshold was available for BMI-normalized handgrip strength. Accordingly, ΔrHGS was also analyzed as a continuous outcome.

### 2.6. Oral Microbiota Assessment

Unstimulated saliva samples were collected at baseline under fasting conditions using sterile saliva collection kits (OMNIgene Oral OM-501, DNA Genotek, Ottawa, ON, Canada). Participants deposited 1 mL of saliva into the collection device. Samples were coded to preserve anonymity and stored according to the manufacturer’s recommendations until analysis.

Bacterial DNA extraction, purification, quantification, amplification of the 16S rRNA gene, sequencing, and bioinformatic processing were performed by ADM Biopolis, University of Valencia (Valencia, Spain), following standardized procedures. Sequencing was performed using the Illumina MiSeq platform. Raw sequencing data were processed using the QIIME2 bioinformatics platform [37]. Amplicon sequence variants (ASVs) were generated after quality filtering and chimera removal using the DADA2 algorithm [38]. Taxonomic assignment was performed against reference microbial databases, including the SILVA database [39,40].

For the present analysis, baseline oral microbiota variables included alpha-diversity indices, specifically Richness, Shannon, and Simpson, together with the relative abundance of selected bacterial genera. These variables were selected to examine whether baseline microbiota characteristics were associated with subsequent changes in rHGS.

### 2.7. Statistical Analysis

Statistical analyses were performed using R statistical software, version 2026.01.2+418 (R Foundation for Statistical Computing, Vienna, Austria). Continuous variables are presented as mean and standard deviation (SD), whereas categorical variables are reported as frequencies and percentages. Baseline characteristics were summarized for the overall sample and compared according to responder status. Continuous variables were compared using independent-samples *t*-tests or Mann–Whitney U tests when appropriate, whereas categorical variables were compared using Fisher’s exact test. Effect sizes for non-parametric group comparisons were estimated using rank-biserial correlation coefficients, as this metric is appropriate for Mann–Whitney U tests. Values range from −1 to +1, with the sign indicating the direction of the group difference and the absolute value reflecting the magnitude of the separation between groups.

Associations between baseline oral microbiota variables and changes in relative handgrip strength (ΔrHGS) were explored using Spearman rank correlation analyses. Differences in alpha-diversity indices and relative abundances of selected bacterial genera between responders and non-responders were evaluated using Mann–Whitney U tests. To address multiple testing in exploratory microbiota analyses, Benjamini–Hochberg false discovery rate (FDR) correction was applied within predefined families of tests, including alpha-diversity indices, genus-level correlations with ΔrHGS, and genus-level comparisons between responders and non-responders. For genus-level group comparisons, FDR correction was applied across all genera meeting the predefined prevalence criterion. Both nominal *p*-values and FDR-adjusted *p*-values are reported where applicable. Findings with nominal *p* < 0.05 but FDR-adjusted *p* ≥ 0.05 were interpreted as exploratory candidate associations.

To explore the association between baseline microbiota variables and ΔrHGS, linear regression models were constructed using ΔrHGS as the dependent variable. Bacterial genus richness was examined as the main microbiota-related predictor. Given the small sample size, individual bacterial genera were not entered simultaneously into a fully adjusted multivariable model. Instead, selected genera showing nominal or near-nominal associations in exploratory analyses were added separately to richness in sensitivity models. These models were considered exploratory and were used to assess whether genus-level associations persisted after accounting for richness. Continuous microbiota predictors were z-standardized before regression; therefore, regression coefficients are reported as unstandardized B coefficients representing the expected change in ΔrHGS per 1-SD increase in the predictor. Regression diagnostics were examined before interpretation of the linear models. Residual normality was assessed using Q–Q plots and Shapiro–Wilk tests, homoscedasticity was evaluated using residual-versus-fitted plots and Breusch–Pagan tests, and influential observations were examined using leverage values, studentized residuals, and Cook’s distance. Given the small sample size, these diagnostics were interpreted descriptively.

Multicollinearity was assessed using variance inflation factors (VIFs) in the regression models including richness and one candidate genus at a time. No evidence of problematic multicollinearity was observed, with all VIF values below 2. Statistical significance was established at *p* < 0.05. Given the exploratory nature of the study and the limited sample size, all findings should be considered preliminary and interpreted as hypothesis-generating.

## 3. Results

The final analytical sample included 18 older adults undergoing exercise training, of whom 15 were women (83.3%). Overall, participants presented a mean age of 65.3 ± 4.5 years and a mean body mass index of 29.0 ± 4.6 kg/m^2^. Sex-related comparisons showed no significant differences in age, body mass index, baseline oral microbiota composition, or alpha-diversity indices (all *p* > 0.05). Men exhibited significantly greater handgrip strength than women (36.58 ± 8.63 vs. 23.99 ± 3.74 kg, effect size = 1.00, *p* = 0.009). Baseline rHGS also tended to be higher in men, although the difference did not reach statistical significance (1.17 ± 0.33 vs. 0.86 ± 0.19, effect size = 0.73, *p* = 0.058). No sex-related differences were identified for post-training rHGS or changes in rHGS (both *p* > 0.05).

**Figure 1:**
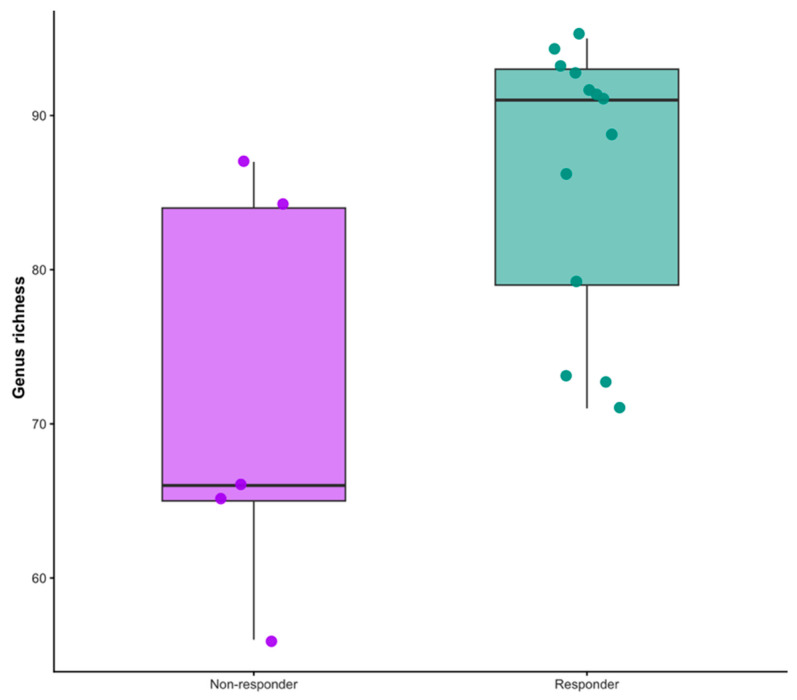
Baseline oral genus richness according to response status.

Table 1 summarizes clinical characteristics and baseline alpha-diversity indices according to rHGS responder status. Responders and non-responders showed comparable age, body mass index, mean handgrip strength, and baseline rHGS values (all *p* > 0.05). As shown in Figure 1, responders showed higher baseline bacterial genus richness than non-responders at the nominal level (86.15 ± 8.90 vs. 71.60 ± 13.32, effect size = −0.72, *p* = 0.023), although this group difference did not remain significant after FDR correction across alpha-diversity indices (*p*FDR = 0.069). No nominal or FDR-adjusted differences were observed for Shannon or Simpson diversity indices.

As shown in Table 2, baseline bacterial genus richness was positively associated with changes in rHGS and remained significant after FDR correction (*ρ* = 0.578, *p* = 0.012, *p*FDR = 0.036). At the nominal level, positive correlations were also observed for Parvimonas and Eubacteriales XIII, whereas Neisseria showed a negative correlation with changes in rHGS. However, these genus-level associations did not remain significant after FDR correction and should therefore be interpreted as exploratory candidate signals. No significant associations were found for Olsenella, Gemella, Shannon index, or Simpson index.

**Figure 2:**
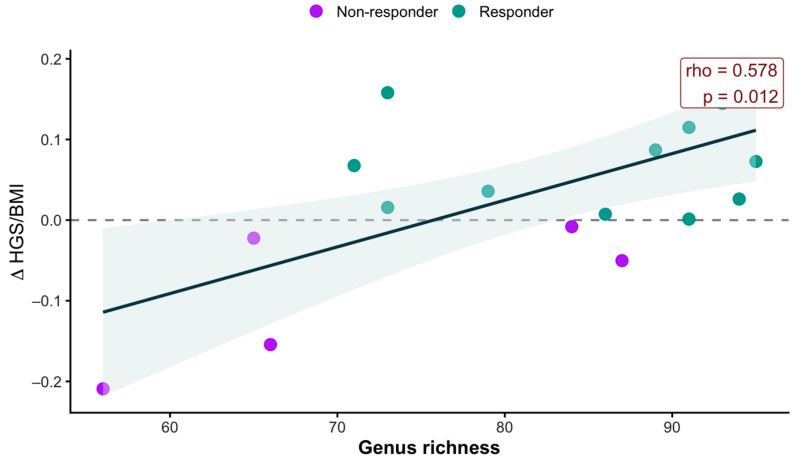
Baseline oral genus richness and training response. The solid line represents the fitted linear regression line, the shaded area indicates the 95% confidence interval, and the horizontal dashed line represents no change in rHGS/BMI.

The association between baseline bacterial genus richness and training-induced changes in rHGS is illustrated in Figure 2. Participants with greater richness tended to exhibit larger improvements in rHGS, whereas lower richness values were generally observed among non-responders.

**Figure 3:**
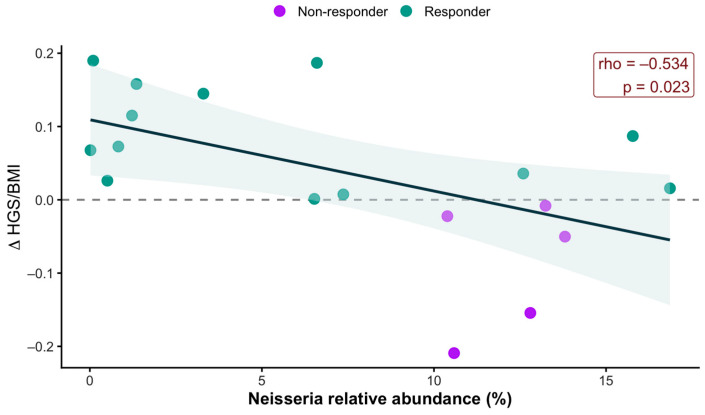
Baseline *Neisseria* abundance and training response. The solid line represents the fitted linear regression line, the shaded area indicates the 95% confidence interval, and the horizontal dashed line represents no change in rHGS/BMI.

The inverse association between baseline *Neisseria* abundance and training-induced changes in rHGS is illustrated in Figure 3. Participants with higher relative abundances of *Neisseria* generally exhibited smaller improvements in rHGS following the intervention, whereas the largest gains were observed among individuals with lower *Neisseria* levels, supporting the negative correlation identified in the bivariate analyses.

Table 3 presents the baseline abundance of selected oral bacterial genera according to training response status. At the nominal level, responders exhibited higher abundances of Eubacteriales XIII (1.047 ± 1.305 vs. 0.152 ± 0.166%, *p* = 0.014), *Parvimonas* (0.862 ± 1.329 vs. 0.110 ± 0.171%, *p* = 0.014), *Olsenella* (0.421 ± 0.915 vs. 0.011 ± 0.020%, *p* = 0.043), and *Gemella* (2.846 ± 1.679 vs. 1.301 ± 0.823%, *p* = 0.049). Conversely, non-responders showed greater abundances of *Neisseria* (12.164 ± 1.574 vs. 5.619 ± 6.033%, *p* = 0.049), while *Haemophilus* displayed a similar pattern without reaching statistical significance (9.219 ± 5.138 vs. 4.157 ± 4.289%, *p* = 0.061). However, none of these genus-level differences remained significant after FDR correction across prevalent genera; therefore, these findings should be interpreted as exploratory candidate associations.

**Figure 4:**
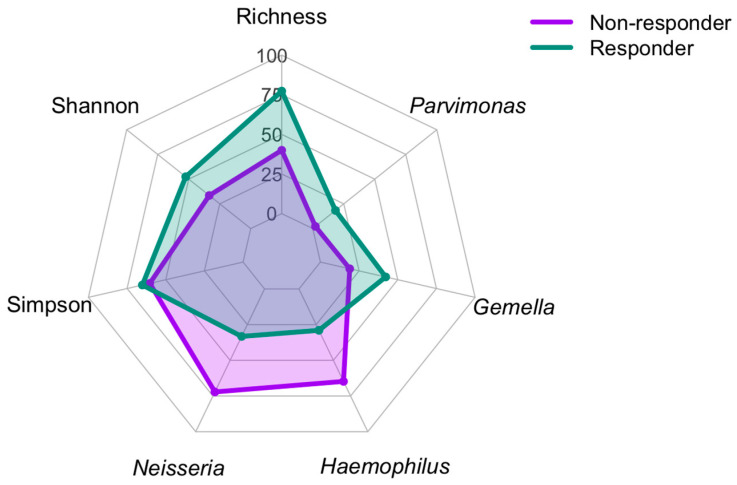
Baseline oral microbiota profile according to training response. Variables were min–max normalized to a common 0–100 scale based on the observed sample range; values represent normalized group means for descriptive comparison.

Figure 4 provides a descriptive visual summary of baseline oral microbiota profiles in responders and non-responders. Responders showed higher normalized values for richness and selected genera identified at the nominal level, whereas non-responders showed higher normalized values for *Neisseria* and *Haemophilus*.

The results of the exploratory linear regression analyses are presented in Table 4. In the primary model, bacterial genus richness was positively associated with changes in rHGS (B = 0.067, 95% CI: 0.025 to 0.109, *p* = 0.004). This association remained significant in sensitivity models in which *Parvimonas*, *Neisseria*, or *Haemophilus* were added separately to richness. In these models, none of the individual genus-level variables showed an independent association with ΔrHGS after accounting for richness. Given the small sample size, these models were interpreted as exploratory and hypothesis-generating, and adjusted R^2^ values are reported descriptively. All exploratory regression models reached overall statistical significance (Model 1: F(1,16) = 11.29, *p* = 0.004; Model 2: F(2,15) = 5.32, *p* = 0.018; Model 3: F(2,15) = 8.13, *p* = 0.004; Model 4: F(2,15) = 5.32, *p* = 0.018).

## 4. Discussion

The present study investigated whether baseline oral microbiota characteristics were associated with inter-individual differences in training-induced changes in relative handgrip strength among older adults. The main finding was that baseline bacterial genus richness was the microbiota-related feature most consistently associated with changes in relative handgrip strength. Richness showed a positive correlation with ΔrHGS that remained significant after FDR correction and also remained associated with ΔrHGS in exploratory sensitivity models in which candidate genera were added separately. By contrast, the difference in richness between responders and non-responders was observed at the nominal level and should be interpreted descriptively. Shannon and Simpson diversity indices were not associated with training response.

These observations suggest that the overall ecological richness of the baseline oral microbiota, rather than general diversity indices or isolated bacterial genera, may be related to variability in strength adaptation in this preliminary cohort. Previous studies have consistently reported substantial heterogeneity in physiological responses to standardized exercise interventions, with some individuals achieving large improvements whereas others experience limited or no measurable adaptations despite similar training exposure [11,13,14]. Growing evidence suggests that microbial ecosystems may contribute to muscle function and physical performance [16,17,18]. In this context, the present findings provide preliminary, hypothesis-generating evidence that baseline oral microbiota characteristics may be associated with exercise responsiveness.

It should also be noted that the exercise intervention was primarily aerobic and performed on cycle ergometers; therefore, handgrip strength was not directly trained. In this context, changes in rHGS should not be interpreted as a direct local adaptation of the upper limb muscles to the training stimulus. Rather, rHGS was used as a practical marker of overall muscle function and functional status in older adults. This interpretation is supported by a meta-analytical review showing small but significant transfer effects of exercise training on handgrip strength in community-dwelling older adults [41]. Therefore, the present findings should be interpreted as associations with a general functional marker rather than as evidence of specific upper-limb strength adaptations induced by cycling-based training.

Although evidence linking oral microbial diversity to exercise responsiveness remains limited, recent population-based studies suggest that oral microbial richness may be associated with healthy ageing and functional status. DeClercq et al. [42] reported that lower salivary microbial richness and phylogenetic diversity were associated with higher frailty levels in middle-aged and older adults, suggesting that oral microbial diversity may reflect biological ageing processes beyond chronological age alone. Similarly, analyses from a large NHANES cohort including more than 4500 middle-aged and older adults from the United States, demonstrated that higher oral microbial richness, assessed through observed ASVs and Faith’s phylogenetic diversity, was independently associated with lower frailty scores after adjustment for multiple confounding variables [43].

This observation is relevant because frailty and impaired physical function share several physiological pathways with age-related declines in muscle strength and exercise capacity. In this context, Azzolino et al. [24] proposed that alterations in oral microbial communities may contribute to frailty and sarcopenia through inflammatory, metabolic, and oral–gut axis mechanisms. Although most mechanistic evidence derives from studies of the gut microbiota, the microbiota–muscle axis provides a plausible biological framework linking microbial composition and diversity with skeletal muscle function [16,17,18]. Potential pathways include immune regulation, chronic low-grade inflammation, energy metabolism, insulin sensitivity, and anabolic signalling processes [16,17]. The mechanisms underlying the associations observed in the present study remain speculative. A richer oral microbial ecosystem may simply reflect greater ecological complexity and stability, characteristics that have been associated with more resilient microbial communities in older adults [44]. Therefore, oral microbial richness should currently be interpreted as a candidate ecological feature associated with exercise responsiveness rather than as a validated biomarker or causal determinant of training adaptation.

The associations observed for individual bacterial genera should be interpreted within the broader ecological context of the oral microbiome. At the nominal level, several genera showed differences according to responder status or associations with ΔrHGS; however, these genus-level findings did not remain significant after FDR correction and should therefore be considered exploratory candidate signals. Oral microbial communities are increasingly recognised as complex ecosystems in which bacterial taxa coexist through dynamic cooperative and competitive interactions rather than acting as independent entities [44,45]. In community-dwelling older adults, genera such as *Neisseria*, *Haemophilus* and *Gemella* have been reported to cluster within specific tongue microbiota profiles, suggesting that changes in the abundance of individual taxa may reflect broader shifts in community structure [44]. From an ecological perspective, the strongest and most consistent signal in the present study was observed for microbial richness. Similarly, studies conducted in athletic populations have shown that exercise and training status are accompanied by coordinated changes in multiple oral bacterial taxa rather than isolated modifications in single genera [26,27,29]. Taken together, the bacterial genera identified in the present study may be better understood as components of broader microbial configurations associated with exercise adaptation rather than as independent determinants of training responsiveness.

Among the genera identified at the nominal level, *Gemella* may be of particular interest. Recent investigations have reported higher relative abundances of *Gemella* species in physically active individuals and competitive athletes, while positive associations with aerobic fitness have also been described [23]. Furthermore, exercise-based interventions in children have been shown to increase the abundance of *Gemella sanguinis* alongside improvements in physical fitness parameters [46]. Although these findings do not support a causal role for *Gemella* in exercise adaptation, they suggest that this genus may be part of microbial configurations associated with favorable physiological profiles. However, the association observed for *Gemella* in the present study did not remain significant after FDR correction and should therefore be interpreted as an exploratory candidate signal.

In contrast, *Neisseria* and *Haemophilus* showed higher relative abundances among non-responders at the nominal level. Both genera are common members of healthy oral microbial communities and have been linked to nitrate metabolism and other physiological functions [23,32]. However, genus-level 16S rRNA sequencing cannot distinguish species, strains, or functional activity, and relative abundance should therefore not be interpreted as direct evidence of nitrate-reducing capacity or other biological functions. Moreover, their relationship with physical performance remains inconsistent across studies and may be strongly influenced by age, oral health status, and overall community structure [45]. In the present study, these genus-level findings did not remain significant after FDR correction and should not be interpreted as independent determinants of exercise responsiveness. Therefore, these taxa are better understood as ecological markers of broader microbial configurations rather than as isolated predictors of training response.

Certain oral bacteria are capable of reducing dietary nitrate to nitrite, which can subsequently be converted into nitric oxide, a molecule involved in vascular function, mitochondrial efficiency, glucose uptake, and skeletal muscle contractility [22,31]. This microbial pathway has attracted growing interest in exercise physiology because oral nitrate-reducing capacity has been associated with markers of cardiorespiratory fitness and exercise performance. In physically active adults, higher oral nitrate-reducing activity has been positively associated with both VO_2_ peak and maximal power output, suggesting a potential link between oral microbial function and exercise-related physiological adaptations [32]. Furthermore, several oral bacterial genera previously linked to nitrate metabolism have been reported to differ according to training status and physical fitness levels [23,31]. Although nitrate-reducing capacity was not assessed in the present study, these studies raise the possibility that functional characteristics of the oral microbiota, rather than taxonomic composition alone, may contribute to inter-individual variability in training responsiveness. Future studies incorporating metagenomic, metabolomic, or functional assessments will be necessary to determine whether nitrate-related microbial pathways contribute to the associations observed in the present cohort.

Most previous studies have examined how exercise influences oral microbiota composition rather than whether baseline microbial characteristics influence training responsiveness. Differences in oral microbial diversity and taxonomic composition have been reported according to training status, sport discipline, and exercise exposure [15,19,26,28], while intervention studies indicate that oral microbial communities are responsive to structured exercise program [27,46]. The present findings extend current knowledge by suggesting that baseline oral microbial profiles may also be associated with the magnitude of training-induced adaptations. Although causality cannot be inferred, this perspective opens new avenues for investigating the biological determinants of exercise responsiveness.

### 4.1. Potential Implications and Future Directions

The potential practical implications of these findings should be interpreted cautiously. Current exercise prescriptions are primarily based on demographic, clinical, and physiological characteristics, whereas biological factors contributing to inter-individual variability in training adaptations remain incompletely understood. In this context, the present findings do not support the use of oral microbiota profiling for individualized exercise prescription at this stage. Rather, if confirmed in larger longitudinal studies, oral microbiota characteristics could be explored as one of several biological factors potentially contributing to variability in exercise responsiveness. This perspective is consistent with the growing interest in personalized medicine, precision nutrition, and individualized exercise prescription [20,27], but its application to oral microbiota profiling remains premature. Furthermore, increasing evidence suggests that oral microbial communities may be modified through behavioral, nutritional, and oral health-related interventions [22,31]. However, whether microbiota-targeted strategies can improve physical function or training adaptation remains unknown. Therefore, these possibilities should be considered future research directions rather than direct clinical implications of the present study.

### 4.2. Limitations

Several limitations should be considered when interpreting the present findings. First, the final analytical sample was small and showed a marked predominance of women. This sex imbalance was partly explained by differential attrition, as all participants excluded from the final analysis were men. These characteristics limit statistical power, increase uncertainty around effect estimates, preclude meaningful sex-stratified analyses, and restrict the generalizability of the findings to more sex-balanced older populations.

Second, the responder/non-responder classification was based on the direction of change in rHGS and should therefore be interpreted with caution. Although a standardized handgrip protocol with three maximal attempts was used to reduce within-session measurement variability, the ΔrHGS > 0 threshold does not account for between-session measurement error or clinically meaningful change. No study-specific standard error of measurement, minimal detectable change, or validated clinically meaningful threshold was available for BMI-normalized handgrip strength. Accordingly, group-based comparisons should be considered descriptive and exploratory, whereas analyses using ΔrHGS as a continuous outcome represent the primary analytical approach.

Third, the exploratory microbiota analyses involved multiple comparisons and regression modelling in a small sample. Although FDR correction was applied and regression models were simplified by adding candidate genera separately to richness, genus-level findings and adjusted R^2^ values should be interpreted with caution. Regression diagnostics did not suggest major departures from residual normality or homoscedasticity; however, some influential or high-leverage observations were detected, further supporting the cautious interpretation of these models. No formal internal validation, such as bootstrap resampling or leave-one-out analysis, was performed; therefore, regression coefficients should be considered exploratory estimates.

Fourth, oral microbiota was characterized using 16S rRNA sequencing, which provides taxonomic information but does not allow direct assessment of microbial function, metabolic activity, nitrate-reducing capacity, or species- and strain-level differences. Consequently, the biological mechanisms underlying the observed associations remain speculative. In particular, functional pathways potentially involved in exercise adaptation, including nitrate–nitrite–nitric oxide metabolism, were not directly evaluated. In addition, post-intervention oral microbiota samples were not analyzed in the present study, which prevents determining whether the exercise program modified oral microbial composition or whether changes in the microbiota were related to changes in rHGS.

Fifth, although the exercise intervention was prospectively conducted, the relationship between baseline oral microbiota and subsequent changes in relative handgrip strength should be considered observational in nature and does not permit causal inference. Several behavioral and clinical variables that may influence both oral microbiota composition and exercise responsiveness were not fully controlled, including oral health status, periodontal disease, dental caries, oral hygiene practices, mouthwash use, habitual diet, nitrate intake, medication use, comorbidities, changes in BMI during the intervention, and detailed adherence or effort during training sessions. These factors may have contributed to residual confounding.

Finally, the present study focused specifically on training-induced changes in relative handgrip strength, although the intervention was mainly aerobic and performed on cycle ergometers. Therefore, handgrip strength was not directly trained, and changes in rHGS should be interpreted as changes in a general functional marker rather than as specific upper-limb strength adaptations. It remains unclear whether similar associations would be observed for other physiological, functional, or performance-related outcomes.

Accordingly, future studies combining larger cohorts, repeated microbiota assessments, functional omics approaches, detailed control of oral health, dietary, behavioral, and clinical variables, and a broader range of exercise outcomes are needed to clarify the role of oral microbiota in exercise adaptation.

## 5. Conclusions

In this preliminary cohort, greater baseline oral microbiota richness was associated with larger improvements in relative handgrip strength following a supervised exercise intervention in older adults. Among the microbiota-related variables examined, bacterial richness showed the most consistent association with training-induced changes in rHGS, whereas genus-level findings were less consistent and should be interpreted as exploratory.

These findings should be considered hypothesis-generating and do not establish clinical applicability or predictive value. Larger longitudinal studies are needed to confirm these observations, with better control of oral health, dietary, behavioral, and clinical confounders, functional microbiome analyses, repeated microbiota assessments, and broader physical performance outcomes.

## Figures and Tables

**Table 1:** Clinical characteristics and baseline alpha-diversity indices according to rHGS responder status.

Variable	Non Responder	Responder	Effect Size	*p*
*n* (%)	5 (27.8)	13 (72.2)		
Age (years)	65.4 (4.4)	65.23 (4.6)	0.11	0.766
BMI (kg/m^2^)	29.78 (5.3)	28.65 (4.4)	0.17	0.622
Mean HGS (kg)	27 (3.4)	25.73 (7.5)	0.32	0.324
Baseline rHGS	0.92 (0.1)	0.91 (0.2)	0.05	0.921
Post rHGS	0.84 (0.2)	1 (0.2)	−0.29	0.375
Genus richness	71.6 (13.3)	86.15 (8.9)	−0.72	0.023
Shannon index	2.68 (0.2)	2.84 (0.2)	−0.48	0.139
Simpson index	0.89 (0.0)	0.9 (0.0)	−0.11	0.767

Values are presented as mean (SD), except *n* (%). Effect sizes correspond to rank-biserial correlation coefficients.

**Table 2:** Correlations between baseline oral microbiota and change in rHGS.

Variable	rho	*p*	*p*FDR
Genus richness	0.578	0.012	0.036
*Parvimonas* (%)	0.575	0.013	0.068
*Neisseria* (%)	−0.534	0.023	0.068
Eubacteriales XIII (%)	0.492	0.038	0.076
*Haemophilus* (%)	−0.455	0.058	0.087
*Olsenella* (%)	0.357	0.146	0.175
*Gemella* (%)	0.267	0.284	0.284
Shannon index	0.257	0.303	0.455
Simpson index	−0.018	0.945	0.945

**Table 3:** Baseline abundance of selected oral bacterial genera in responder and non-responder to training.

Genus	Non-Responder	Responder	Effect Size	*p*	*p*FDR
Eubacteriales XIII (%)	0.152 (0.166)	1.047 (1.305)	−0.78	0.014	0.423
*Parvimonas* (%)	0.11 (0.171)	0.862 (1.329)	−0.78	0.014	0.423
*Olsenella* (%)	0.011 (0.02)	0.421 (0.915)	−0.65	0.043	0.423
*Neisseria* (%)	12.164 (1.574)	5.619 (6.033)	0.63	0.049	0.423
*Gemella* (%)	1.301 (0.823)	2.846 (1.679)	−0.63	0.049	0.423
*Haemophilus* (%)	9.219 (5.138)	4.157 (4.289)	0.60	0.061	0.431

**Table 4:** Exploratory linear regression models for changes in rHGS according to baseline oral microbiota variables.

Model	Predictor	B	95% CI	*p*	Adjusted R^2^
Model 1: richness	Bacterial genus richness	0.067	(0.025, 0.109)	0.004	0.377
Model 2: richness + *Parvimonas*	Bacterial genus richness	0.068	(0.02, 0.116)	0.009	0.337
Model 2: richness + *Parvimonas*	*Parvimonas*	−0.003	(−0.045, 0.04)	0.894	0.337
Model 3: richness + *Neisseria*	Bacterial genus richness	0.054	(0.011, 0.096)	0.017	0.456
Model 3: richness + *Neisseria*	*Neisseria*	−0.006	(−0.014, 0.001)	0.088	0.456
Model 4: richness + *Haemophilus*	Bacterial genus richness	0.064	(0.005, 0.124)	0.036	0.337
Model 4: richness + *Haemophilus*	*Haemophilus*	−0.001	(−0.013, 0.011)	0.883	0.337

Note: Regression coefficients are unstandardized B coefficients from models using z-standardized microbiota predictors; therefore, they represent the expected change in ΔrHGS per 1-SD increase in the predictor.

## Data Availability

The data presented in this study are available on request from the corresponding author due to the signed consent agreements around data sharing, which only allow access to external researchers for studies following the project’s purposes.

## Abbreviations

The following abbreviations are used in this manuscript:

ASV	Amplicon Sequence Variant
BMI	Body Mass Index
CI	Confidence Interval
DNA	Deoxyribonucleic Acid
HGS	Handgrip Strength
rHGS	Relative Handgrip Strength
NHANES	National Health and Nutrition Examination Survey
NO	Nitric Oxide
QIIME2	Quantitative Insights Into Microbial Ecology 2
SD	Standard Deviation
16S rRNA	16S Ribosomal Ribonucleic Acid
